# Epidemiological, clinical, and therapeutic characteristics of Behçet's disease: a monocentric study in Tunisia

**DOI:** 10.11604/pamj.2021.40.13.19146

**Published:** 2021-09-03

**Authors:** Fatma Daoud, Imène Rachdi, Mehdi Somai, Anissa Zaouak, Houda Hammami, Meriem Ouederni, Rym Maamouri, Hana Zoubeidi, Molka Tougorti, Jihène Ksouri, Besma Ben Dhaou, Zohra Aydi, Samy Fenniche, Monia Cheour, Fatma Boussema

**Affiliations:** 1 Internal Medicine Department, Habib Thameur Hospital, Faculty of Medicine of Tunis, University Tunis El Manar, Tunis, Tunisia,; 2 Department of Dermatology, Habib Thameur Hospital, Faculty of Medicine of Tunis, University Tunis El Manar, Tunis, Tunisia,; 3 Ophthalmology Department, Habib Thameur Hospital, Faculty of Medicine of Tunis, University Tunis El Manar, Tunis, Tunisia

**Keywords:** Behçet's disease, vasculitis, treatment, prognosis

## Abstract

**Introduction:**

to describe the epidemiological, clinical, therapeutic and evolving characteristics of Behçet´s disease and identify prognostic factors.

**Methods:**

we have realized a retrospective, single-center study, conducted over a period of 26 years and including 130 patients presenting Behçet´s disease and hospitalized in an Internal Medicine Department.

**Results:**

the mean age of the Behçet´s disease at onset was 30.3 ±8.8 years and that at diagnosis was 34.6 ±9.4 years. The sex ratio (male/female) was 2.5. The mean delay of diagnosis was 53.5 months. Oral aphthosis was constant. The frequency of the manifestations was: genital aphtosis 71.5%, pseudofolliculitis 84.6%, erythema nodosum 11.5%, positive pathergy test 50%, ocular disease 36.9%, venous thrombosis 30%, arterial disease 4.6%, joint damage 30.8%, neurological disease 19.2% and digestive disease 0.8%. The male gender was significantly associated with ocular involvement (p =0.02), venous disease (p =0.01) and occurrence of relapses (p =0.01). The mean follow up was 68.5 ± 77.3 months. The poor survival prognostic factors were male gender, ocular involvement, venous disease, cardiovascular disease, a duration of follow up ≤12 months and a diagnostic delay ≤ 24 months. **Conclusion:** improving the prognosis of Behçet´s disease requires a shortening of the time to diagnosis, multidisciplinary collaboration, intensive treatment of functional threats, regular monitoring, and patient adherence.

## Introduction

Behçet’s disease (BD) is a systemic vasculitis characterized by buccal and/or genital aphthosis with or without systemic manifestations. Its prognosis depends on clinical forms. Although BD seems to be the most common form of vasculitis in Tunisia [1], no representative studies have focused on the characteristics of BD population and its prognostic factors. Due to its frequency in the country, its diagnostic and therapeutic difficulties and its threatening prognosis, we conducted this study. The aims were to describe the epidemiological, clinical, therapeutic characteristics, evolution modalities, and prognostic factors of Tunisian patients with BD.

## Methods

**Type of the study**: this study is a single-center, retrospective, descriptive and longitudinal study. We retained all patients with BD admitted to an Internal Medicine Department in Tunisia over a period of 26 years.

### Patients

**Inclusion criteria**: we retained all patients with confirmed BD responding to the International Criteria for BD [2]. A positive diagnosis of BD was retained only after having at least four points of the clinical criteria: a) **aphtosis of the mouth (two points)**; b) **genital aphtosis (two points)**; c) **skin damage (one point)**: pseudofolliculitis, erythema nodosum, papulo-pustular lesions and/or acneiform nodules not explained by corticosteroids and cutaneous aphtosis. d) **Positive pathergic test (one point)**: it was considered positive if an aseptic papuleor pustule (superior than 2 mm) was objectified at the point of the bite. e) **Eye damage (two points)**: in case of ocular abnormalities in ophthalmic examination, ophthalmological exploration was supplemented with retinal fluoresce in angiography. Visual field, visual evoked potentials and/or the ocular echography were practiced according to the clinical context.

We included: **major ocular lesions**: uveal involvement (anterior uveitis, intermediate uveitis or hyalitis, posterior uveitis and panuveitis) and/or retinal vasculitis (RV). **Minor ocular lesions**: conjunctival ulcerations, keratoconjunctivitis, episcleritis and / or scleritis.

### Optic neuropathies

**Complications and ocular sequelae**

**Vascular involvement (One point)**: it was confirmed by the imaging data. We included **venous thrombosis**: superficial vein thrombosis, deep vein thrombosis (DVT), pulmonary embolism (PE) and /or cerebral venous thrombosis (CVT). **Arterial injury**: thrombosis and/or arterial aneurysms.

**Neurological impairment (one point)**: retained in front of somatic neurological examination, neuroimaging and / or cerebro-spinal fluid.

We included: a) parenchymatous lesions retained on the cerebro-medullary magnetic resonance imagery sequences by hypo-signal lesions in T1 sequence and in hyper-signal in T2 and T2 flair sequence; b) **non-parenchymal lesions**: they included CTV, arterial thrombosis and/or cerebral arterial aneurysms; **neurological sequelae**: objective neurological disorders persisting during the evolution and that could be a source of neurological handicap.

**Non inclusion criteria**: we have not included the patients with confirmed BD but had never been hospitalized for BD and the patients to whom the diagnosis was rejected during the evolution and whose medical files were lost and/or unworkable.

**Modalities of evaluation**: we defined different modalities of evaluation: a) a patient lost sight of was defined by a follow-up period necessarily longer than a month. b) A patient lost sight of out of hand was defined by a duration of follow-up obligatorily lower than one month. c) An improvement or remission was defined by subjective and/or objective good evolution of the clinical signs with a complete and/ or partial remission. d) A complete remission was defined by a complete resolution with disappearance of any sign of the disease, in the absence of any relapse during the follow-up period. e) A partial remission was defined by a significant but incomplete decrease in the signs of the disease, while maintaining a risk of subsequent relapse during the observation period. f) A relapse was defined by the reappearance of clinical signs after a favourable evolution. g) A stabilization was defined by a stability that no longer progressed toward improvement or worsening with a risk of relapse during the follow-up period. h) An aggravation was defined by an exacerbation of clinical lesions with appearance of complications.

**Statistical analysis**: all data were analysed by the software SPSS 19.0. Statistical analysis contained absolute frequencies and relative frequencies. We calculated averages, medians and standard deviations and we determined the extreme values for the quantitative variables. For the analytic study, the comparison between two averages was made with the Student test for independent series, and by the non-parametric test of Mann and Whitney in case of low effectives. The comparison in percentages on independent series were made by the chi-Square test of Pearson and in case of significance of chi-square test and its non-validity, with exact bilateral Fisher test. The study of the binding was carried out by the Kaplan Meier method. Survival prognostic factors was performed in univaried analysis by comparing survival curves with Log rank test. In all statistical tests, the level of significance is 0.05.

## Results

**Epidemiological characteristics**: we included 130 patients. Disease incidence was of five new cases per year. Patients were Tunisian in 97.7% of the cases and Algerian in two cases. The mean age at first disease manifestation was 30.3 years ± 8.8 (extremes 12-58 years). The mean age at diagnosis was 34.6 years ± 9.4 (extremes 15-65 years). Five patients were aged less than 16 years. Sex ratio (M/F) was 2.5.

### Clinical study

**Onset disease**: buccal aphthosis was inaugural in 55.4% of the cases. Genital and buccal aphthoses were associated in 25.4% of the cases. Severe organ involvement was revealed in 27 BD cases (20.8%). Mean diagnosis delay was 53.5 months ± 65.2. Mean diagnosis delay for 72 patients with inaugural buccal aphthosis was 58.1 months ± 79.6 and 49.5 months ± 60.5 for other patients.

### Clinical manifestations

**Cutaneous mucosal manifestations**: buccal aphthosis was constantly observed among patients. Evolutive genital aphthosis was observed in 71.5% of the cases. Genital scars were noted in 45.4% of patients. Two patients had peri-analaphthosis. Skin hypersensitivity was noted in 46.9% of the cases. Pathergy test was performed in 87.7% of cases and was positive in 50% of the cases. Skin damages were observed in 87.7% of the cases. Pseudofolliculitis and erythema nodosum (EN) were observed in 84.6% and 11.5% of the cases, respectively.

**Ocular manifestations**: they were observed in 36.9% of the cases and inaugural in 10.8% of the cases. Ocular involvement was discovered in 6.2% of the cases. Complications and sequelae were diagnosed in respectively 47.9% and 6.2% of the cases. Patients were definitely blind at the moment of diagnosis in 8.3% of the cases. Uveal involvement was the most frequent lesion (77.1%) followed by retinal vasculatis (62.5%). Hyalitis was diagnosed in 15 patients (31.3%) and was associated to posterior uveitis in six cases. Seven patients (14.6%) had retinal hemorrhage.

**Vascular manifestations**: they were noted in 31.5% of cases. It was about 35 cases of venous manifestations in 35 cases, arterial manifestations in two cases and both in four cases. Deep venous thrombosis(DVT) was the most frequent vascular manifestations (78%).

**Venous manifestations**: venous impairment was observed in 95.1% of vascular manifestations and 30% of BD patients. It revealed the diagnosis in 20.7% of the cases. Venous manifestation appeared during BD evolution in 9.2% of cases with a mean delay of 45.8 months ± 45.9. There were DVT in 32 cases and superficial vein thrombosis in 32 cases. The locations of DVT were the lower limbs (84.4%), the vena cava (28.1%), the upper limbs (9.4%), pulmonary embolism (PE) (9.4%), cerebral venous thrombosis (6.2%) and Budd Chiari syndrome (3.1%).

**Arterial impairment**: it was observed in 14.6% of vascular manifestations and 4.6% of patient with BD and revealed BD in three cases. All patients were male. Arterial manifestations were as follows: isolated arterial aneurysms (n=1), isolated arterial thrombosis (n=4), and both manifestations (n=4). Pulmonary artery was the most frequent disease localization (n=5).

**Neurological manifestations**: central nervous system disorders was reported in 19.2% and was inaugural in two cases. They were caused by parenchymatal lesions (88%). Psychiatric manifestations were observed in 36% of the cases with neuro-BD (6.9% of patients with BD).

**Cardiac manifestations**: they were inaugural in one case. Three patients had pericardial effusion and coronary involvement.

**Other manifestations**: joint damage and digestive disease were diagnosed in respectively 30.8% and 0.8% cases.

**Biology**: biological inflammatory disorders were observed in 77 patients (59.2%). HLA typing was performed in 47 patients (32.2%) and was positive in 48.9% of the cases. Only one patient had a combined deficiency of proteins S and C.

### Behcet´s disease treatment

**Figure 1:**
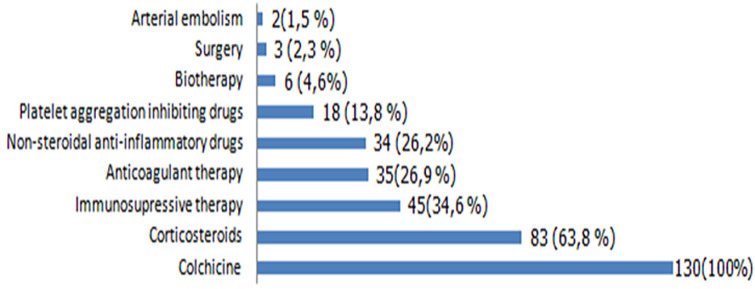
therapeutic means in Behçet´s disease (results n (%))

**Therapeutic means in Behcet´s disease**: the different therapies used for BD are summarized in Figure 1.

**Anticoagulant therapy and platelet aggregation inhibitors**: all patients with DVT and /or arterial thrombosis were treated with anticoagulant therapies. The mean period of treatment was 60.9 months with extremes 2 to 218. Two patients had hemorrhagic complications secondary to vitamin K overdose.

**Evolution modalities**: twenty-two patients (16.9%) were lost sight of the out of hand. The mean follow-up was 68.5 ± 77.3 months (5.7 years), and extremes were 6 days to 26 years. Among 130 patients, 48.5% noted an improvement. It was about vascular manifestations (53.6%), cutaneous and mucosal manifestations (46.9%) and ocular manifestations (25%). Complete and partial remission were observed in respectively 26.2% and 22.3%. Disease stabilization and a worsening were observed in respectively 28.5 and 4.6%. The mortality rate in our study was 1.5%. A fatal vasculo-BD occurred in two cases. The relapse was observed in 42.3%. The mean number of relapses per patient during BD evolution was 7 ± 6. The maximum number of relapses was 14. The mean period between BD diagnosis and first disease relapse was 34 months ± 53. Definitive sequelae were observed in 10% of cases (n=13) and were mainly secondary to ocular and neurological involvement.

### Analytic study

**Figure 2:**
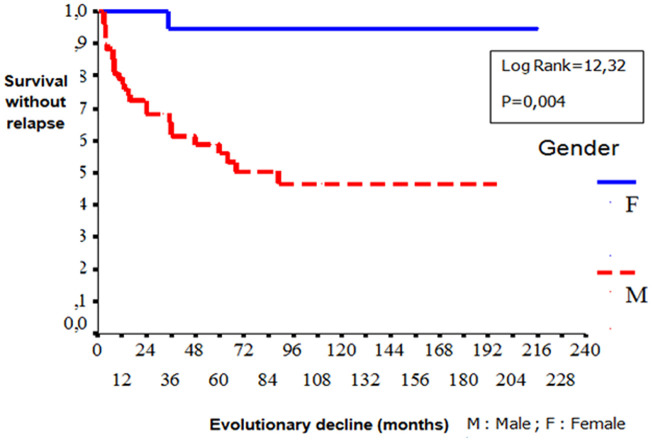
comparison curves of survival without relapses and without aggravation

**Figure 3:**
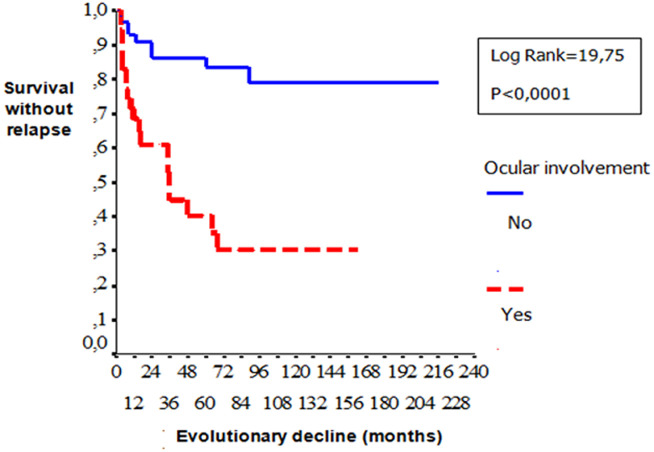
comparison curves of survival without relapses and without aggravation according to the presence or not of ocular involvement

**Figure 4:**
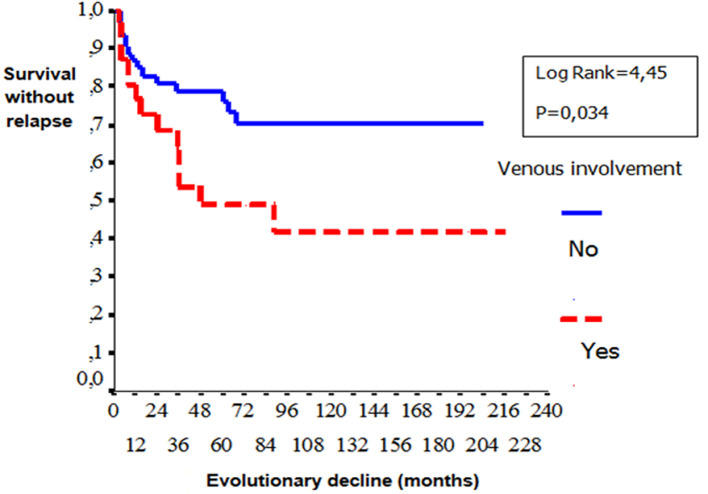
comparison curves of survival without relapses and without aggravation according to the presence or not of venous involvement

**Figure 5:**
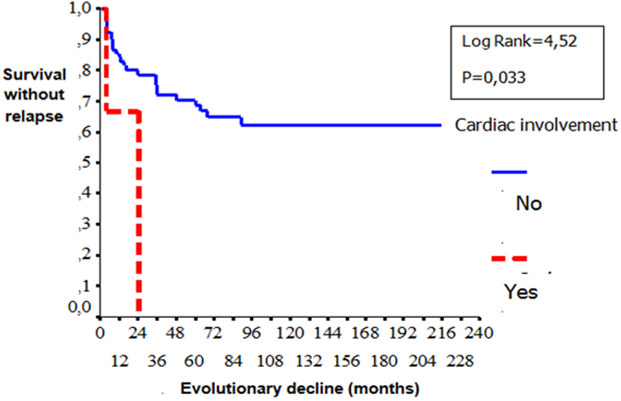
comparison curves of survival without relapses and without aggravation according to the presence or not of cardiac involvement

**Gender**: women developed significantly more frequently EN (p=0.03) than men. Male gender was significantly associated with ocular involvement (p=0.02), venous manifestations (p=0.01), corticosteroid treatment (p=0.007), immunosuppressive therapy (p=0.01), anticoagulation (p=0.009) and relapses (p=0.001). Table 1 shows the comparison of the patients in terms of gender: a) **Age**: no difference in epidemiological, clinical, biological, diseaseevolution and prognosis in terms of age was observed among patients. b) **Types of manifestations**: patients with ocular involvement developed less frequent biological inflammatory disorders (p=0.001) and vascular manifestations (p=0.016). Remission is rarely observed compared with patients without ocular BD (p=0.001). Biological inflammatory disorders were more frequently observed in patients with angio-BD (p=0.003). Patients with articular manifestations develop significantly less relapses than patient with other organ involvement. (p=0.007). Table 2, Table 3 and Table 4 show the comparison of the patients in terms of the ophthalmology and vascular and articular manifestations of the BD, respectively. c) **HLA profile**: no difference in the type of manifestations and HLAB51 profile was observed among the patients. d) **Prognostic factors**: two cases of death were noted in our series. We were unable to identify prognostic factors of mortality. We studied the survival events of patients without relapses and survival events without aggravation and thus released the prognostic factors of survival that can influence the evolutionary profile and the survival of these patients. We compared the two subgroups of patients: 1) **group1 (n = 74)**: patients who had no relapses or worsening of the disease (n=51) and patients who had only isolated mucocutaneous relapses (n=23); **group 2 (n = 29)**: patients who have relapsed ocular, vascular, articular, and neurological manifestations and patients who have had a worsening of their disease. According to Kaplan Meier analysis, one- three- five-10-and 20 years the survival rates with-neither relapses nor worsening were observed in respectively 84%, 70%, 67%, 61% and 61% of the cases. The disease was stabilized after 8 years of evolution (Figure 2). These prognostic factors are summarized in Figure 3, Figure 4, Figure 5.

**Table 1:** socio-demographic characteristics of mothers/husbands who had children 6-23 months of age (n=414) in Finote Selam town, April 2017

Characteristics(Variable)	Categories	Frequency (n=414)	Percentage
**Age of mothers' (in years)**	<20	21	5.1
	20-24	99	23.9
	25-29	105	25.4
	30-34	113	27.3
	≥35	76	18.4
**Marital status**	married	377	91.1
	Unmarried/ Divorced/ Widowed	37	8.9
**Religion**	Orthodox	388	93.7
	Muslim	26	6.3
**Mothers' educational**	No education	165	39.9
	Primary education (1-8)	115	27.8
**Status**	Secondary and above education(9+)	134	32.3
**Mothers' occupation**	House worker/unemployed	250	60.4
	Employed	164	39.6
**Radio owners'**	Yes	315	76.1
	No	99	23.9
**Television owners'**	Yes	303	73.2
	No	111	26.8
**Monthly income (ETB)**	<999	97	23.4
	1000-1999	107	25.8
	2000-2999	107	25.8
	3000-3999	58	14.1
	≥4000	45	10.9
**Mothers fasting status**	Yes	313	75.6
	No	101	24.4
**Currently, breastfeed**	Yes	376	90.8
	No	38	9.2
**Sex of child**	Male	201	48.6
	Female	213	51.4
**Child's age(months)**	6-11	110	26.6
	12-17	125	30.2
	18-23	179	43.2
**Fathers' educational status**	No education	128	30.9
	Primary education(1-8)	108	26.1
	Secondary education (9-12)	105	25.4
	Above secondary	73	17.6
**Fathers' occupation**	Government worker	144	34.8
	Private business	135	32.6
	Daily labor	82	19.8
	Farmer	53	12.8

Notes: ETB, Ethiopian birr

**Table 2:** comparison of the patients according to the ophthalmologic manifestations of Behcet´s disease

Evolutives and clinical biologic characteristics	G1 N=48 n (%)	G2 N=82 n (%)	P
Buccal aphthosis	48(100)	82(100)	*
Genitalaphthosis	32(66,7)	61(74,4)	NS
Pseudofolliculitis	44(91,7)	66(80,5)	NS
Erthyemanodusum	5(10,4)	10(12,2)	NS
Positive pathergy test	17(41,5)	40(54,8)	NS
Articular manifestations	12(25)	28(34,1)	NS
Vascular manifestations	9(18,8)	32(39)	**0,016**
Neurologic manifestations	9 (18,8)	16(19,5)	NS
Cardiacmanifestations	1(2,1)	3(3,7)	NS
HLA B 51 positive	9(60)	14(43,8)	NS
Biologicinflammatory syndrome	9(18,8)	48(58,5)	**0,001**
Remission	12(25)	51(62,2)	**0,001**
Aggravation	5(10,4)	1(1,2)	NS
Relapses	24(50)	31(37,8)	NS

* : No statistics are calculatedbecause oral aphthosisis a constant; G1: Group with ocular involvement; G2: Group withoutocularinvolvement; NS: Not significant

**Table 3:** comparison of the patients according to the vascular manifestations of Behcet's disease

Evolutives and clinical_biologiccharacteristics	G1 N=41 n(%)	G2 N=89 n (%)	P
Buccal aphthosis	41(100)	89(100)	*
GenitalAphthosis	31(75,6)	62(69,7)	NS
Pseudofolliculitis	32(78)	78(87,6)	NS
Erthyemanodusum	5(12,2)	10(11,5)	NS
Positive pathergytest	21 (56,8)	36(46,8)	NS
Articular manifestations	9(22)	39(43,8)	0,016
Vascular manifestations	12(29,3)	28(31,5)	NS
Neurologic manifestations	10(24,4)	15 (16,9)	NS
Cardiac manifestations	4(9,8)	0(0)	0,009
HLA B 51 positive	7(46,7)	16(50)	NS
Biologicinflammatory syndrome	32(78)	25(28,1)	0,003
Remission	2(4,9)	0(0)	NS
Aggravation	22 (53,6)	41(46,1)	NS
Relapses	13(31,7)	42(47,2)	NS

* : No statistics are calculatedbecause oral aphthosisis a constant; G1: Group with vascular involvement; G2: Group without vascular involvement; NS: Not significant

**Table 4:** comparison of the patients according to the articular manifestations of Behcet´s disease

Evolutives and clinical biologic characteristics	G1 N=40 n (%)	G2 N=90 n(%)	P
Buccal aphthosis	40(100)	90(100)	*
Genitalaphthosis	29(72,5)	64(71,1)	NS
Pseudofolliculitis	34(85)	76(84,4)	NS
Erthyemanodosum	5(12,5)	10(11,1)	NS
Positive pathergy test	19 (54,3)	38(48,1)	NS
Articular manifestations	12(30)	36(40)	NS
Vascular manifestations	12(30)	29(32,2)	NS
Neurologic manifestations	9(22,5)	16(17,8)	NS
Cardiac manifestations	2(5)	2(2,2)	NS
HLA B 51 positive	8(44,4)	15(51,7)	NS
Biologicinflammatory syndrome	20(50)	37(43,6)	NS
Remission	24(60)	37(41,1)	NS
Relapses	5(12,5)	50(55,5)	**0,007**

* : No statistics are calculatedbecause oral aphthosisis a constant; G1: Group with articular involvement; G2: Group withoutarticularinvolvement; NS: Not significant.

## Discussion

Our study was conducted during a long period of 26 years. A few works have been focused on BD characteristics and survival factor prognosis without relapse nor worsening. A better knowledge of different manifestations and prognosis factors of BD, are a key for early diagnosis and better management to improve the function and prognosis and guarantee a better quality of life for North African patients. However, retrospective and single center characteristics were the limitations of this study. Selection biais can also explain the important prevalence of multisystemic forms. BD seems to be the most frequent vasculitis in Tunisia [1]. In a North African study conducted in 2009, 674 Tunisian patients with BD from the north and the central regions of Tunisia were examined [3]. BD incidence in our study was five new cases per year. BD affects young patients and rarely those older than 50 years [4]. Mean age at BD diagnosis vary from 29.1 to 38 years [1, 5]. Our study matched the results found in the literature with a mean age diagnosis at 34.6 years ± 9.4. An important male BD prevalence is noted in our study. Similar results were noted in North Africa and Middle East. These results vary according to studies and countries [3, 6]. The sex ratio tend to decline in Turkey, Japan and Korea [7-9]. Familial forms of BD were reported in 9.2% of cases compared with 15.4% in Korea [10], 21.9% in Turkey [11], and 3% in Morocco [12].

Cutaneous-mucosal manifestations were inaugural in many studies [6, 13], and the same results were observed in our study. The mean diagnosis delay was the same in Japan [8], France [5] and Greece [7], and it was later compared in Brazil [14]. This difference is explained in our case by late consultations and diagnostic difficulties. Clinical manifestation distribution varies from one study to another. This fact may be explained by population heterogeneity and clinical criteria for each manifestation. Buccal aphthosis was observed in all patients in our study, which is in concordance with literature results [15]. Tunisian patients more frequently develop pseudofolliculitis than Koreans [10] and Turkish [16] and less frequently nodular fever than Turkish [16] and Iranians [17]. Male gender was significantly associated with oculo-BD in our study. Association between oculo-BD and HLA B51 positivity was reported in the literature [8]. This fact was not demonstrated by our study. Angio-BD was negatively correlated with ocular involvement (p=0.016) and this result is in concordance with literature observations [3, 9]. The important venous thrombosis incidence and scarcity of arterial manifestations during BD progression were observed in our study and other studies [18]. Our study also confirm lower limb and vena cava predilection of thrombosis [1, 18]. Neuro-BD incidence in the literature varies from 2.3% to 44% of the cases [19]. It depends on ethnicity, methods of recruitment and isolated headache inclusion. In spite of neuro-behçet´s clinical polymorphism, parenchymatous involvement was more frequent than extra parenchymatous form as observed in other studies [20, 21].

Central nervous system involvement in BD is characterized by diffuse involvement with brain stem predilection observed in 21.4% to 70%of the cases. Hemispherical and medullary localizations were observed in respectively 14% to 19.8% and in 2.6% to 14% of the cases [22, 23]. The results of our study agree with the data from the medical literature. Brain stem localization was noted in 64% of the cases. No medullary localization was observed. Meningoencephalitis was observed in three quarters of the patients with neuro-BD according to data from the medical literature [19]. Parenchymatous form was also the most characteristics localization with encephalitis predominance (56%). The frequency of pseudotumoral form (8%) [21, 23], and aseptic meningitis (4%) [1] was comparable to the results of previous studies. However, optical neuritis was relatively more frequent in our study (16%) [24-26]. Cerebral magnetic resonance imaging is described as the gold standard in neuro-BD management and medical supervision [24, 25]. This fact was confirmed by our study. Cerebral magnetic resonance imaging detected abnormalities in 96% of the cases compared with 28% for brain scan.

Cardiac manifestations incidence was comparable to the literature [1, 12]. Cardiac involvement and DVT are frequently associated in our study such as Geri *et al*. [26]. Pericardial effusion was the most frequent cardiac manifestation in our study.This observation was also reported in the literature [1, 17, 27]. The main objective treatments for BD are to reduce disease flare prevalence and avoid angio- and oculo-BD sequelae. No consensus in BD management was reached. Treatment depends on organ involvement and disease severity [28, 29]. In our study, different therapeutic protocols were conductedaccording to the clinical manifestations of BD, evolutivity and patients´ socio-economic conditions. The last fact explains the limited use of biological treatments. As described in the literature, treatments based on colchicine, non-steroidal anti-inflammatory drugs, and topical corticosteroids were sufficient as described in literature to control cutaneous and articular manifestations [28]. However, aggressive treatment with immunosuppressive therapy is needed in some cases with severe or refractory forms. The EULAR recommendations for the management of BD were revised and they developed five overarching principles and 10 recommendations related to the different types of organ and system involvement in BD [29, 30].

## Conclusion

BD has an important polymorphism and a relatively high incidence in the Maghreb. Poor survival prognostic factors are male gender, ocular involvement, venous disease, cardiovascular disease, a duration of follow up ≤12 months and a diagnostic delay ≤24 month. Behind the therapeutical progress, prognosis improvement is very important by shortening the diagnosis delay, multidisciplinary staff participation, and intensive treatment of the functional involvement and regular monitoring of patients. Education is important for the treatment of BD to guarantee patients´ compliance.

### What is known about this topic

- BD is an inflammatory, systemic, chronic disease that evolves by relapses;
- The clinical presentation is polymorphic and associated with a bucco-genital aphthosis with various systemic manifestations. The positive diagnosis is often difficult especially in the absence of aphthosis;
- The therapeutic management remains controversial, due to the scarcity of therapeutic trials and the absence of standardized criteria for measuring the evolution. Its evolution is unpredictable. Its prognosis varies according to its clinical presentation. The neurological and ocular manifestations are responsible for a progressive deterioration of the functional prognosis. The vascular and digestive disorders condition the vital prognosis.

### What this study adds

- The male gender is significantly associated with ocular involvement, venous disease and occurrence of relapses;
- The poor survival prognostic factors are male gender, ocular involvement, venous disease, cardiovascular disease, a duration of follow up ≤12 months and a diagnostic delay ≤24 months.
